# Loss of Sugar Detection by GLUT2 Affects Glucose Homeostasis in Mice

**DOI:** 10.1371/journal.pone.0001288

**Published:** 2007-12-12

**Authors:** Emilie Stolarczyk, Maude Le Gall, Patrick Even, Anne Houllier, Patricia Serradas, Edith Brot-Laroche, Armelle Leturque

**Affiliations:** 1 Centre de Recherche des Cordeliers, Université Pierre et Marie Curie-Paris6, UMR S 872, Paris, France; 2 Université Paris Descartes, UMR S 872, Paris, France; 3 INSERM, U872, Paris, France; 4 French National Institute for Agricultural Research (INRA), AgroParisTech, UMR914 Nutrition Physiology and Ingestive Behavior, CRNH-IdF, Paris, France; University of California at Los Angeles, United States of America

## Abstract

**Background:**

Mammals must sense the amount of sugar available to them and respond appropriately. For many years attention has focused on intracellular glucose sensing derived from glucose metabolism. Here, we studied the detection of extracellular glucose concentrations *in vivo* by invalidating the transduction pathway downstream from the transporter-detector GLUT2 and measured the physiological impact of this pathway.

**Methodology/Principal Findings:**

We produced mice that ubiquitously express the largest cytoplasmic loop of GLUT2, blocking glucose-mediated gene expression *in vitro* without affecting glucose metabolism. Impairment of GLUT2-mediated sugar detection transiently protected transgenic mice against starvation and streptozotocin-induced diabetes, suggesting that both low- and high-glucose concentrations were not detected. Transgenic mice favored lipid oxidation, and oral glucose was slowly cleared from blood due to low insulin production, despite massive urinary glucose excretion. Kidney adaptation was characterized by a lower rate of glucose reabsorption, whereas pancreatic adaptation was associated with a larger number of small islets.

**Conclusions/Significance:**

Molecular invalidation of sugar sensing in GLUT2-loop transgenic mice changed multiple aspects of glucose homeostasis, highlighting by a top-down approach, the role of membrane glucose receptors as potential therapeutic targets.

## Introduction

Sensing of sugar is a survival mechanism enabling organisms to know when to constitute and mobilize tissue energy stores. It is known that glucose homeostasis relies on appropriate detection of glucose concentration, but glucose-sensing mechanisms remain poorly understood in mammalian cells. Intracellular glucose is sensed and the resulting signal relayed by metabolic messengers in tissues. Glucokinase and mitochondrial oxidative fluxes (ATP/ADP ratio) are involved in regulation of ATP-sensitive K+ channels controlling insulin secretion and are well-described glucose sensors in pancreatic ß cells [1]–[3]. Intracellular glucose metabolism stimulates sensitive-gene transcription in the liver [4], [5], [6]. Intracellular glucose metabolism is a common feature of mammalian cells and modulation of glucose sensing by metabolic inhibitors, although potent, is not recommended *in vivo* as most cell functions and vital parameters would be affected.

Yeasts detect extracellular sugar concentrations with proteins located in the external membrane [7], including sugar receptors of the GPCR family and transporter-detectors of the sugar transporter family [8]. In adipocytes, the glucose transporter GLUT1 detects glucose with its C-terminus domain activating the ERK pathway by a mechanism independent of glucose transport and metabolism [9]. In pancreatic beta cells, investigations have yield conflicting results concerning the possible role of detector for GLUT2 in glucose-induced insulin secretion. The group of B. Thorens demonstrated that in mice GLUT2 participates to the first phase of insulin secretion [10]. Unexpectedly, Fanconi-Bickel patients bearing GLUT2 invalidating mutations do not develop overt diabetes [11] but their insulin secretions were not investigated. This suggests that GLUT2 is less involved in insulin secretion in human than in mice. Importantly, the role of GLUT2 in insulin synthesis has not been fully characterized yet in mice and man.

In hepatic cells, GLUT2 detects glucose and activates a signaling pathway through its large cytoplasmic loop leading to glucose-induced transcription, independent of glucose metabolism [12]. The sodium-glucose transporter homolog SGLT-3, was identified as a glucose sensor in the plasma membrane of enteric neurons triggering membrane depolarization [13]. Due to these plasma membrane detectors, cells can adapt to changes in extracellular sugar concentrations. The role of glucose detectors in glucose homeostasis has not been evaluated *in vivo*. Such a study should provide information on the importance of the glucose-sensing pathway triggered by detectors, and help elucidate the role of plasma membrane therapeutic targets in modulation of glucose homeostasis.

GLUT2 is expressed in various tissues involved in glucose homeostasis; thus, we investigated the physiological significance of GLUT2 detection of extracellular glucose concentration. GLUT2 is involved in metabolic glucose sensing by mediating bi-directional glucose transport, adjusting intracellular glucose concentration. It is also involved in the detection of sugar abundance at the plasma membrane *in vitro* [12]. GLUT2 has been shown to contribute to control of food intake and secretion of insulin and glucagon in GLUT2-null mice [14], [15], [16], but detection of both intracellular and extracellular glucose concentrations as well as transport are invalidated in those mice.

We quantified, *in vivo*, the physiological contribution of extracellular sugar detection independent of changes in glucose metabolism. For this purpose, we used a molecular tool that blocks glucose-induced gene expression *in vitro*, but preserves glucose metabolism [12]. We generated transgenic mice expressing the GLUT2-loop domain to prevent the detection of extracellular sugar abundance. We report here the impact of the loss of extracellular glucose detection on various aspects of glucose homeostasis.

## Results

### Generation of GLUT2-loop transgenic mice

**Figure 1:**
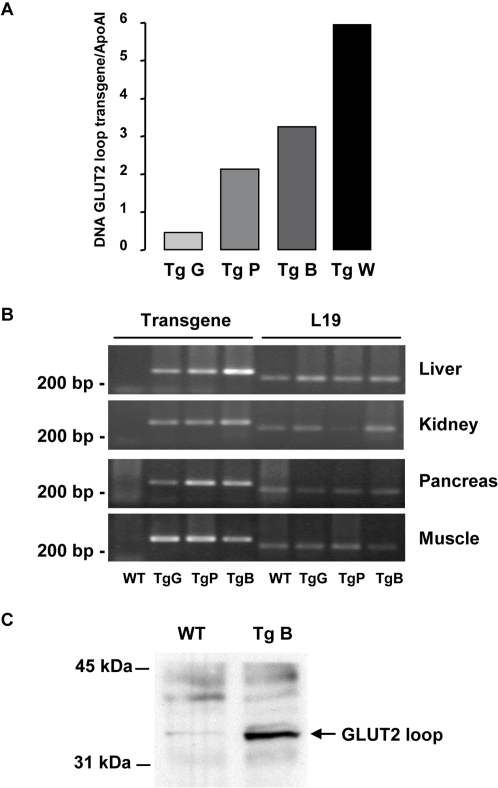
Generation of GLUT2-loop transgenic mice. A: Quantification of the transgene copy number in genomic DNA from independent lines of mice (Tg G, P, B and W) to the reference gene Apolipoprotein A1 (ApoA1). B: RT-PCR analysis of transgene and L19 control mRNA levels in various tissues. C: Immunoprecipitation and immunoblot analysis showing the presence of GLUT2 loop in liver homogenate from transgenic mice.

We generated transgenic mice expressing the large cytoplasmic GLUT2 loop to investigate its sugar detection function *in vivo*. We obtained four transgenic mice with various copy numbers of transgene (Fig. 1A). Mouse ‘W’ had the highest copy number of transgene, growth failure and urinary glucose loss, but survived at weaning and did not develop diabetes. Unfortunately, ‘W’ was sterile. Founder ‘B’ had a high transgene copy number and was viable but some homozygous did not survive. Heterozygous and homozygous mice from ‘P’ and ‘G’ founders were viable.

Transgene mRNA was present in all the tissues tested, as expected from the ubiquitous actin promoter used, but at various levels (Fig. 1B). Transgene mRNA expression was absent in wild-type (WT) mice. There was no clear correlation between cDNA copy number and mRNA levels of the transgene. We detected the protein coded by the transgene by immunoprecipitation of liver extracts with a specific antibody raised against the GLUT2 intracellular loop (Fig. 1C).

Thus, we created three lines of transgenic mice expressing various amounts of the transgene in GLUT2- and non GLUT2-expressing tissues.

### Invalidation of extracellular sugar detection in GLUT2 expressing tissues

**Figure 2:**
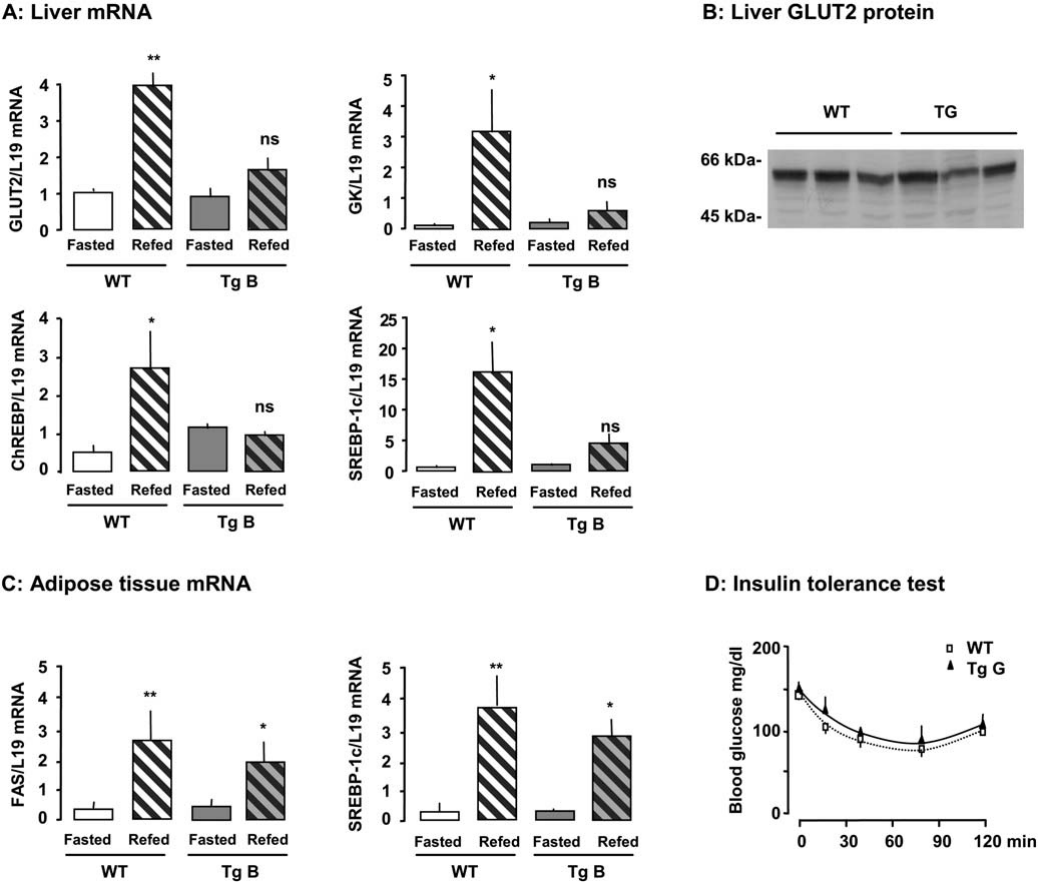
Impairment of extracellular sugar detection in GLUT2-expressing tissues in transgenic mice. Effect of a glucose-rich diet on gene expression in liver (A) and adipose tissue (C). Transgenic (Tg) and wild-type (WT) mice were fasted for 48 h and refed for 15 h before liver and epididymal fat pad biopsies. Levels of mRNA were analyzed by real time PCR. Values are presented as means±S.E.M. (n = 3 to 5 mice/group). Statistical differences between refed and fasted mice are indicated by *P<0.05, **P<0.01, and ns non significant. B: GLUT2 protein levels in total membrane preparations from the liver of mice fed a glucose-rich diet for five days. D: Blood glucose concentrations during an insulin tolerance test in wild-type and transgenic mice. Values are presented as means±S.E.M (n = 8 to 10 mice/group).

We have previously shown *in vitro* that the GLUT2 loop blocks glucose stimulation of sensitive gene transcription. In this study, we investigated *in vivo* the role of the GLUT2 loop. For this purpose, we fed mice with a standard diet, then either fasted the mice for 48h or refed (after fasting) them a glucose-rich diet. We studied genes stimulated directly by glucose and genes stimulated indirectly by insulin secreted in response to glucose ingestion (GLUT2, ChREBP, glucokinase, SREBP-1c). The mRNA coded for these genes accumulated in the liver of wild-type mice 15h after they consumed a glucose-rich diet, as expected (Fig. 2A). However, these mRNAs remained at basal levels in transgenic mice (Fig. 2A), despite similar food consumption. *In vivo* expression of the GLUT2 loop, therefore, sets the gene-expression profile in fed transgenic mice to the level established in fasted wild-type mice.

GLUT2 protein levels in liver membrane were similar in wild-type and transgenic mice fed a glucose-rich diet for five days (Fig. 2B). Thus, the phenotype of transgenic mice in which glucose detection by GLUT2 is impaired is not due to low GLUT2 protein levels in liver membranes. This feature is related to liver expression of the transgene inhibiting detection of glucose abundance.

We assessed the specificity of GLUT2-mediated sugar detection, compared to other GLUTs. For this purpose, we studied tissues that do not express GLUT2 but other members of the GLUT family. We detected transgene mRNA in epididymal adipose tissue (not shown), as in muscle (Fig. 1B). Feeding a glucose-rich diet induced a similar level of accumulation of FAS, SREBP-1c (Fig. 2C) and ACC mRNAs (not shown) in epididymal fat pads from wild-type and GLUT2-loop transgenic mice. These findings show that the expression of the GLUT2 loop did not affect GLUT4-expressing tissues and suggest that other glucose-sensing pathways are involved in these tissues. Moreover, peripheral tissues of transgenic and wild-type mice had similar insulin sensitivity, as determined by an insulin tolerance test (Fig. 2D). These findings indicate that the GLUT2 loop did not interfere with peripheral glucose disposal by muscle and adipose tissues that express GLUT4.

Thus, we produced transgenic mice in which GLUT2-mediated glucose detection was specifically abolished.

### Preference for lipid oxidation

**Figure 3:**
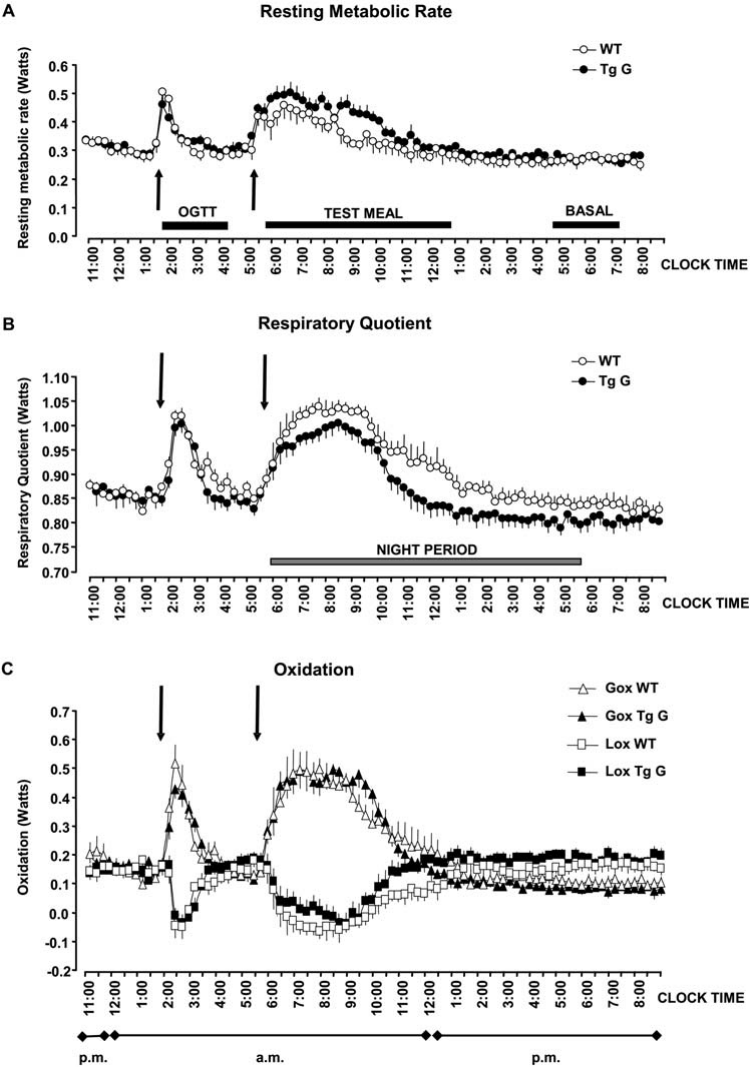
Energy expenditure in mice after impairment of extracellular sugar detection. Progression of resting metabolism rate, respiratory quotient and calculated oxidation recorded at ten-second intervals in wild-type (WT) and transgenic (Tg) mice. The test meal was given just before the lights were turned off and the response measured until metabolism rate and respiratory quotient returned to pre-meal levels. The changes in resting metabolism rate and respiratory quotient were included in the calculation of the changes in glucose (Gox) and lipid (Lox) oxidation.

We investigated the adaptation of mice to impaired detection of glucose abundance by measuring the energy expenditure resulting from glucose and lipid oxidation (Fig. 3). We determined the time course of resting metabolic rate and respiratory quotient averaged at 15-minute intervals in wild-type and transgenic mice (Fig. 3A and B). Resting metabolism, respiratory quotient, and glucose and lipid oxidation (Fig. 3C) were similar in wild-type and transgenic mice before food consumption.

The peak response for metabolic rate during oral glucose load of 3.6 mg/g (OGTT) occurred after ten minutes and the peak response for respiratory quotient occurred after 30 minutes. There was no significant difference between groups (p = 0.269). Accordingly, there were no differences between groups for glucose and lipid oxidation during oral glucose load. The overall metabolic rate and overall respiratory quotient (measured by the areas under the respective curves) were slightly, but not significantly lower (p = 0.178) in transgenic mice than in wild-type mice during oral glucose load.

Then, we fed mice a test meal of 1g standard laboratory chow containing sugars and lipids. The difference in the thermogenic response between the two groups was amplified (Fig. 3). Pre-meal metabolic rate, respiratory quotient, and glucose and lipid oxidation were similar in wild-type and GLUT2-loop transgenic mice. The peak response of the respiratory quotient to the meal was slightly smaller in amplitude and occurred three hours later in transgenic mice than in wild-type mice. The overall increase in metabolic rate during the test meal was significantly larger in GLUT2-loop mice than in wild-type mice (3.2±0.3 versus 2.0±0.2 kJ, p<0.02), but the increase in respiratory quotient was smaller in transgenic mice than in wild-type mice (Fig. 3A and B). This phenomenon resulted from a lower level of post-meal inhibition of lipid oxidation in transgenic mice than in wild-type mice; post-meal glucose oxidation was not different between groups (Fig. 3C).

We estimated the basal metabolic rate between 5:00 and 7:30 a.m. at the end of the calorimetry session when the mice were in a post-absorptive/fasting state. Basal metabolic rate was similar between groups, but respiratory quotient was slightly lower in transgenic mice (0.8W) than in wild-type mice (0.84W) (Fig. 3B). We calculated that lipids fueled 60% of basal metabolism in wild-type mice and 70% in transgenic mice.

Thus, GLUT2-loop transgenic mice had lower levels of glucose oxidation than wild-type mice but higher levels of lipid oxidation.

### Attenuated response to low and high glucose levels

**Figure 4:**
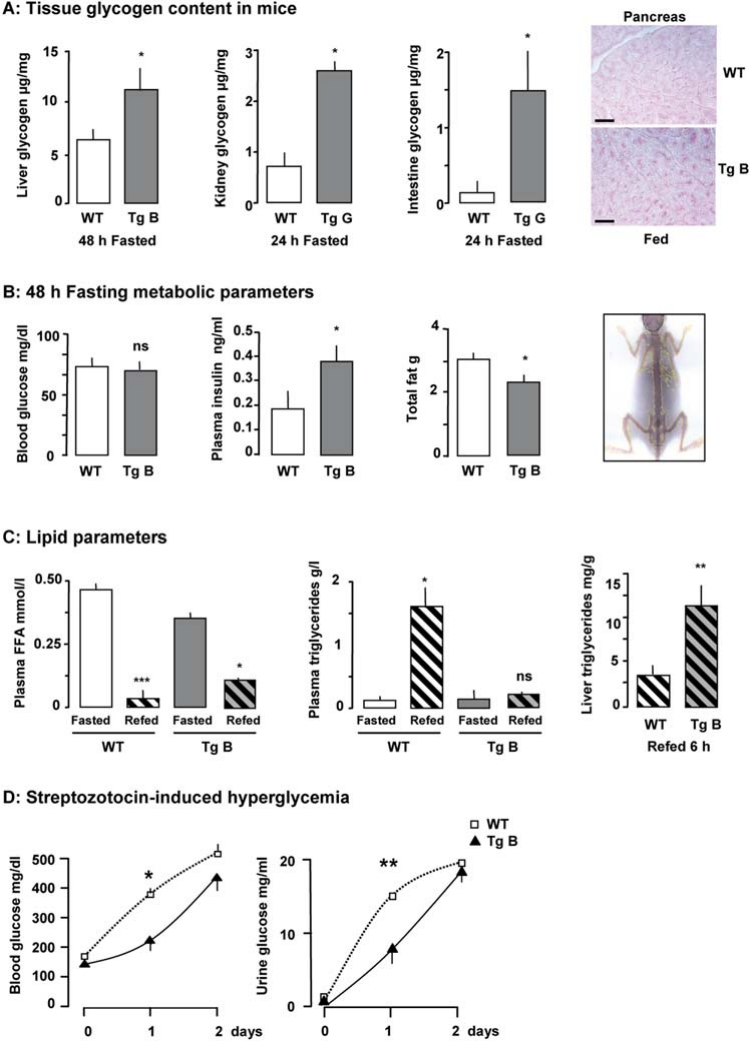
Detection of low and high glucose levels. A: Left panel: glycogen content in liver, kidney and intestine from fasting wild-type (WT) and transgenic (Tg) mice. Values are presented as means±S.E.M. (for the liver n = 6 to 7 mice per group, for the kidney and intestine n = 3 mice/group). Statistical differences between transgenic and wild-type mice are indicated as *P<0.05. Right panel: Representative pancreas sections from fed wild-type and transgenic mice stained with PAS reagent. The black bar corresponds to 50 µm. B: Fasting metabolic parameters. Blood glucose concentration, plasma insulin concentration and total fat are indicated for wild-type and transgenic mice after fasting for 48 h. Values are presented as means±S.E.M. (n = 4 mice/group). Statistical differences between transgenic and wild-type mice are indicated as *P<0.05 and ns non significant. Right panel: Example of a DEXA scan. C: Lipid metabolism. Plasma free-fatty acids (FFA) and triglyceride concentrations in wild-type and transgenic mice in the fasting state or 6h after refeeding. Values are presented as means±S.E.M. (n = 3 to 5 mice/group). Statistical differences between refed and fasted mice are indicated as ***P<0.001, *P<0.05 and ns non significant. Right panel: Liver triglyceride content in liver extract from wild-type and transgenic mice 6h after refeeding. Values are presented as means±S.E.M. (n = 4 to 5 mice/group). Statistical differences between transgenic and wild-type mice are indicated as **P<0.01. D: Blood glucose and urine glucose levels measured after steptozotocin injection. Values are presented as means±S.E.M. (n = 3 to 4 mice/group). Statistical differences between wild-type (open squares) and transgenic (closed triangles) mice are indicated as *P<0.05, **P<0.01.

We tested the capacity of mice with impaired GLUT2-mediated sugar sensing to detect sugar deficit. After 48h of fasting, the glycogen content in liver of wild-type mice was lower than taht of GLUT2-loop transgenic mice (Fig. 4A). Residual glycogen stores were larger after fasting in liver, kidney and intestine of transgenic mice than of wild-type mice (Fig. 4A), possibly resulting from the slight but constant lower rate of glucose oxidation in fasted GLUT2-loop transgenic mice (Fig. 3C). This phenomenon occurred despite normal blood glucose concentration (Fig. 4B), but was probably associated with higher plasma insulin level in transgenic mice than in wild-type mice (Fig. 4B). Total body fat content was lower in transgenic than in wild-type fasted mice (Fig. 4B). This finding may be related to the higher level of fat oxidation observed in the fasted transgenic mice (Fig. 3C).

Plasma free fatty acids (FFA) levels were high in fasted wild-type mice, and plasma triglyceride levels were low, as expected (Fig. 4C). Plasma FFA levels were lower and plasma triglyceride levels were higher six hours after refeeding (Fig. 4C). We observed a similar, but attenuated profile for plasma FFA levels in transgenic mice, but plasma triglyceride levels were not significantly different in refed and fasted transgenic mice. Consequently, the triglyceride content remained high in the liver of fed transgenic mice (Fig. 4C).

This finding indicates that GLUT2-loop transgenic mice are more resistant to fasting than wild-type mice and suggests that transgenic mice do not properly detect sugar deficit.

Injection of streptozotocin (a drug that poisons pancreatic ß-cells) led to pronounced hyperglycemia. We observed significantly less pronounced hyperglycemia and glucosuria in transgenic mice than in wild-type mice (Fig. 4D) the day after drug injection. However, the protection against streptozotocin-induced diabetes was transient, as blood and urine glucose levels in the two groups of mice were the same two days after drug injection (Fig. 4D).

### Glucose tolerance and pancreatic functions

**Figure 5:**
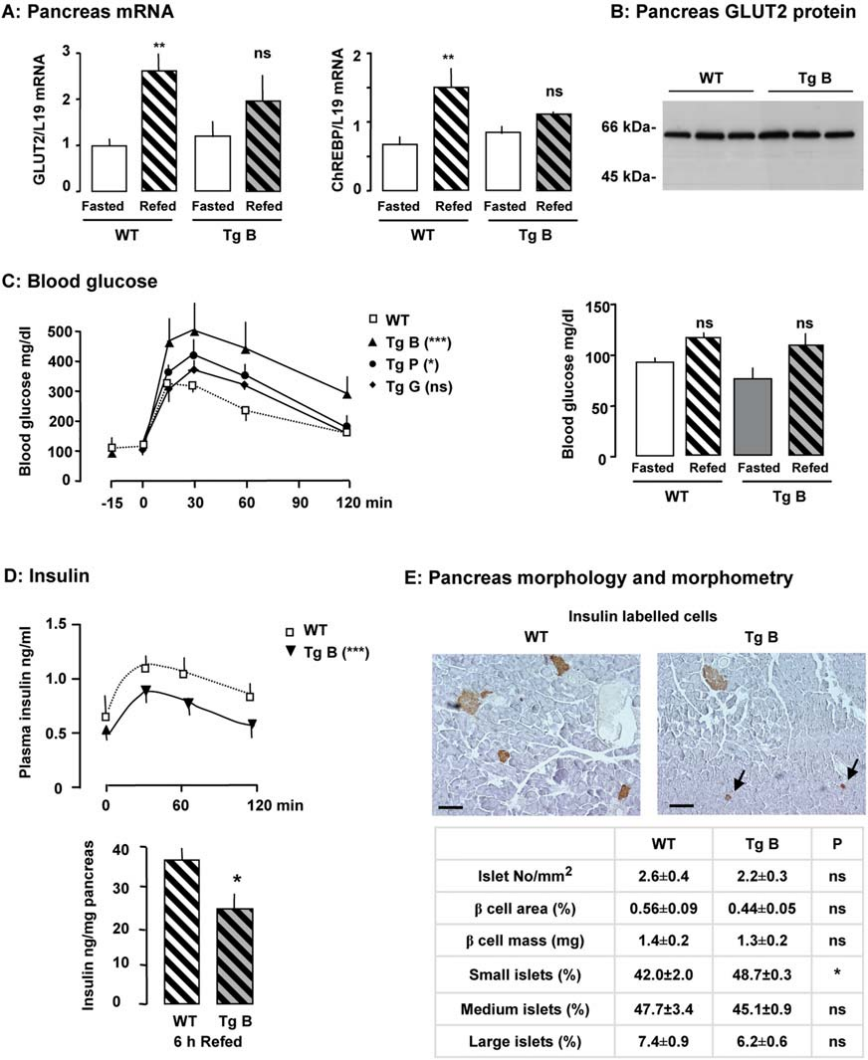
Pancreatic function in mice after impairment of extracellular sugar detection. A: Effect of a glucose-rich diet on gene expression in the pancreas of wild-type (WT) and transgenic (Tg) mice fasted for 48h and refed for 15 h. Levels of mRNA were analyzed by real-time PCR. Values are presented as means±S.E.M. (n = 3 to 4 mice/group). Statistical differences between refed and fasted mice are indicated as **P<0.01 and ns non significant. B: GLUT2 protein levels in total membrane preparations of pancreas from mice fed a glucose-rich diet for five days. C: Left panel: Blood glucose concentrations during an oral glucose tolerance test in wild-type and transgenic mice fasted for 24 h (n = 17 for wild-type mice, n = 2 to 5 for transgenic mice). Statistical differences between transgenic and wild-type mice are indicated as ***P<0.001, *P<0.05 and ns non significant (two-way ANOVA) for the areas under the curves. Right panel: Blood glucose concentrations in wild-type and transgenic mice in the fasted state or 6 h after being refed with a glucose-rich diet. Values are presented as means±S.E.M. (n = 4 to 8 mice/group). D: Upper panel: Plasma insulin concentrations during an oral glucose tolerance test in fasted wild-type and transgenic mice (n = 10 to 13 mice/group). Statistical differences between transgenic and wild-type mice are indicated as ***P<0.001 (two-way ANOVA) for the areas under the curves. Lower panel: Pancreatic insulin content in mice 6h after being refed a standard diet. Values are presented as means±S.E.M. (n = 5 mice/group). Statistical differences between transgenic and wild-type mice are indicated as *P<0.05. E: Upper panel : Representative immunostaining with antibody against insulin of pancreatic sections from wild-type and transgenic mice. Arrows indicate small islets. The bar corresponds to 100 µm. Lower panel: Histomorphometric comparisons of islet number, size and ß-cell mass. Proportion of small islets (<25 µm) to total number of islets is statistically different indicated as *P<0.03 between transgenic and wild-type mice.

Glucose modulates many pancreatic functions. We verified that the regulation of a subset of glucose-sensitive genes was impaired in the pancreas of GLUT2-loop transgenic mice (Fig. 5A). In the pancreas, mRNA of GLUT2 and ChREBP (Fig. 5A) and glucokinase (not shown) accumulated significantly in wild-type but not in transgenic mice, 2 h after an oral glucose load. We observed similar accumulation of ACC mRNA in the two groups of mice (not shown). These findings suggest that glucose detection in the pancreas, as measured by the stimulation of target genes, is altered in transgenic mice. GLUT2 protein levels were similar in pancreas membrane preparations from wild-type and transgenic mice fed a glucose-rich diet for five days (Fig. 5B).

Blood glucose concentrations during an oral glucose tolerance test remained significantly higher in transgenic mice than in wild-type mice, indicating glucose intolerance (Fig. 5C). This occurred despite glucose and lipid oxidation having the same kinetics in the two groups of mice (Fig. 3C). Nevertheless, we observed glucose intolerance in all three transgenic mouse lines (Fig. 5C). Six hours after being refed a glucose-rich diet, blood glucose concentrations were similar in the two groups of mice (Fig. 5C). This indicates that after a delay transgenic mice can regulate blood glucose concentration similarly to wild-type mice.

Plasma insulin levels during the glucose load remained significantly lower in GLUT2-loop transgenic mice than in wild-type mice (Fig. 5D). Thus, a defective insulin response to oral glucose may explain, at least in part, glucose intolerance. Pancreatic insulin content was 30% lower in transgenic mice than in wild-type mice six hours after being refed a glucose-rich diet (Fig. 5D). Impairment of glucose detection by GLUT2 in the pancreas attenuated changes in insulin production in response to glucose or to diet.

Insulin production is regulated by glucose at various steps. Proinsulin mRNA accumulation was 2.9±0.5 times higher in wild-type and 2.3±0.3 times higher (p = 0.3) in transgenic mice fed a high-glucose diet for two hours than in fasted control mice. These findings together with the lower insulin protein content in pancreas of transgenic mice suggest that a GLUT2-mediated glucose detection step is involved in the translation or maturation of insulin.

We therefore analyzed the morphology of pancreatic islets. Immunohistochemical and morphometrical analyses showed that total islet number/mm^2^ of transgenic mice was not different from that of wild-type mice (Fig. 5E). However, the proportion of insulin-labeled cells organized as small islets (<25 µm) was higher in pancreas of GLUT2-loop transgenic mice than of wild-type mice (p = 0.03) (Fig. 5E). This finding is consistent with the lower pancreatic insulin content in GLUT2-loop transgenic mice than in wild-type mice six hours after being refed (Fig. 5D).

The invalidation of GLUT2-mediated glucose detection in pancreatic ß-cells impaired insulin production, but did not modify GLUT2 protein level and insulin mRNA accumulation. Though islet morphology appeared preserved, insulin-positive cell organization was altered in transgenic mice.

### Excretion of excess glucose by the kidney

Besides the insulin action on peripheral tissues, excess glucose is efficiently cleared from blood by renal excretion. We thus analyzed the role of GLUT2-mediated extracellular glucose detection in kidney of transgenic mice.

**Figure 6:**
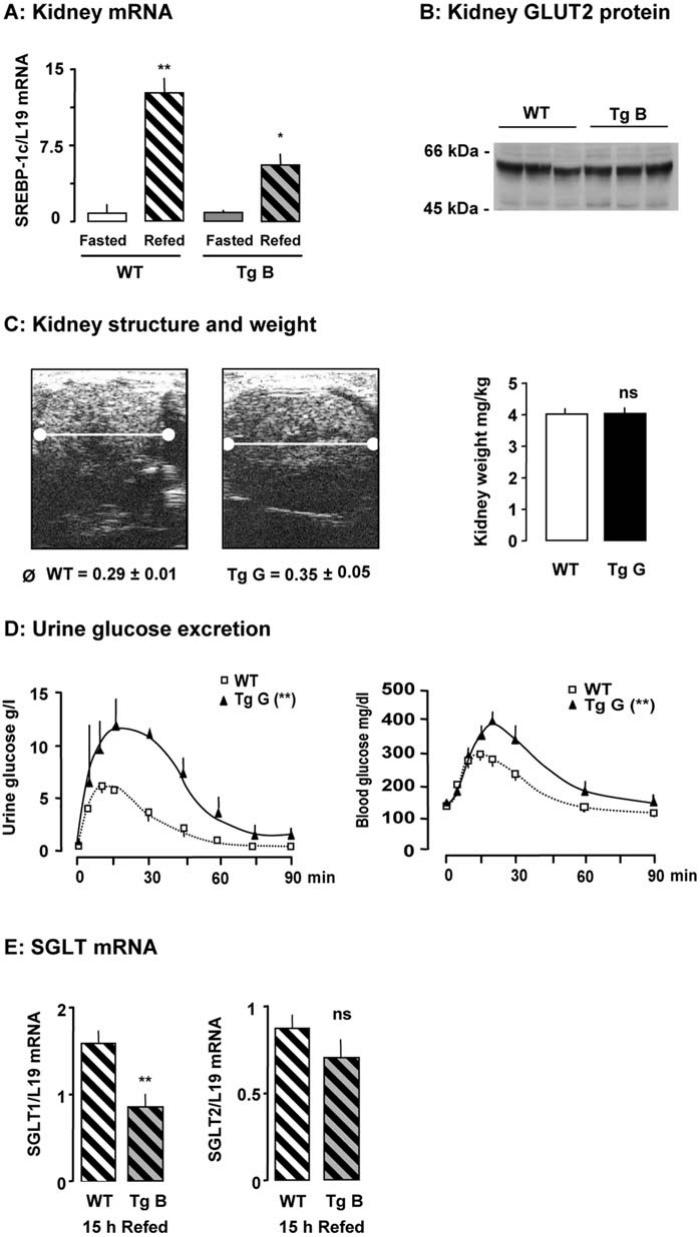
Kidney function in mice after impairment of extracellular sugar detection. A: Effect of a glucose-rich diet on gene expression in the kidney of transgenic (Tg) and wild-type (WT) mice fasted for 48 h and refed for 15 h. Levels of mRNA were analyzed by real-time PCR. Values are presented as means±S.E.M. (n = 3 to 4 mice/group). Statistical differences between refed and fasted mice are indicated as *P<0.05, **P<0.01 and ns non significant. B: GLUT2 protein levels in total membrane preparations of kidney from mice fed with a glucose-rich diet for five days. C: Structure, size and weight of kidneys from wild-type and transgenic mice shown by ultrasonic image (transverse cross section). D: Urine and blood glucose concentrations during an oral glucose tolerance test in fasted wild-type and transgenic mice (n = 3 mice per group). Statistical differences between transgenic and wild-type mice are indicated as **P<0.01 (two-way ANOVA) for the areas under the curves. E: Levels of SGLT mRNA were analyzed by real-time PCR. Values are presented as means±S.E.M. (n = 3 to 4 mice/group). Statistical differences between refed and fasted mice are indicated as **P<0.01 and ns non significant.

We verified that GLUT2-mediated sugar detection was impaired in kidney of transgenic mice. Unlike in wild-type mice, consumption of a glucose-rich diet after 48 h of fasting did not lead to accumulation of GLUT2 and SREBP-1c mRNAs in transgenic mice (Fig. 6A and not shown). Basal levels of these mRNAs were not different in kidney of wild-type and transgenic mice and the GLUT2 protein levels in membrane preparations from wild-type and transgenic mice were the same (Fig. 6B). The lack of glucose-stimulated gene expression confirmed that kidney of transgenic mice did not properly detect extracellular glucose concentration.

We assessed the consequences of impaired GLUT2-mediated glucose detection in the kidney by examining kidney structure by echography. The kidneys of transgenic mice were 160% larger in diameter than wild-type kidneys (Fig. 6C), although they had similar weights. We observed a difference in echogenicity due to glycogen but not to water accumulation in transgenic kidney (Fig. 6C and 3A).

The ratio of urine volume excreted to the water volume consumed per day was identical in the two groups (not shown). We analyzed the urine composition and found that the measured parameters did not differ between the two groups if mice were fed a standard diet (Table 1). In contrast, the consumption of a glucose-rich diet led to significantly greater urine glucose loss in GLUT2-loop transgenic mice than in wild-type mice (Table 1). We used an oral glucose load to document this specific loss of glucose in the urine. The transgenic mice lost substantially more glucose in the urine than wild-type mice did (Fig. 6D). Moreover, we detected glucose in the urine of wild-type mice when the blood glucose concentration reached 200±10mg/dl, whereas glucosuria appeared as soon as the blood glucose concentration reached 170±4mg/dl in transgenic mice (p<0.03) (Fig. 6D). This indicates a lower glucose threshold in transgenic mice than in wild-type mice. We observed a significantly lower SGLT1 mRNA level in transgenic mice than in wild-type mice 15 hours after being refed, whereas SGLT2 mRNA levels were similar in the two groups. These findings suggest that impairment of glucose detection provoked major adaptation in glucose renal reabsorption.

**Table 1:** Urinary excretion in mice after impairment of extracellular sugar detection. Metabolic and electrolyte levels in 24-h urine samples from wild-type (WT) or transgenic mice fed a standard or a glucose-rich diet. Values are presented as means±S.E.M. (n = 6 per group). Statistical differences between wild-type and transgenic mice are indicated as *P<0.05 ns non significant, nd indicates not determined.

	Standard diet			Glucose-rich diet		
Parameters	Wild-type	Transgenic	P	Wild-type	Transgenic	P
Glucose (g/mmol creatinine)	0.033±0.007	0.023±0.004	ns	0.016±0.005	0.067±0.021	*
K (mmol/mmol creatinine)	25±2	23±2	ns	15±2	13±2	ns
Cl (mmol/mmol creatinine)	98±7	110±8	ns	108±8	99±10	ns
Ca (mmol/mmol creatinine)	1.9±0.4	3.1±0.8	ns	0.6±0.1	1.8±0.6	ns
Na (mmol/mmol creatinin)	42±5	46±6	ns	n.d.	n.d.	
Pi (mmol/mmol creatinin)	nd	nd		16±2	16±2	ns

## Discussion

In this study, we show that prevention of sugar detection strongly affects many mechanisms involved in glucose homeostasis. Transgenic mice excreted massive amount of glucose in urine, which protected them against excess glucose despite attenuated pancreatic insulin production and a preference for lipid oxidation over glucose oxidation. The GLUT2 loop, used here as a molecular tool, blocked the sugar detection triggered by GLUT2 without affecting basal mRNA levels in liver, kidney and pancreas. On the other hand, the GLUT2-null mice, in which both sugar entry and sugar detection are invalidated, deteriorates after weaning, and mice die probably because they are unable to handle excess glucose by appropriate insulin secretion [10], [15]. The phenotype of GLUT2-loop transgenic mice may be related to the maintenance of glucose- and insulin-sensitive functions at basal levels.

The expression of the GLUT2-loop transgene interfered with GLUT2-triggered glucose detection. This highlights the efficiency of this plasma membrane chemoreceptor for glucose, and provides a new therapeutic target for glucose homeostasis. Consumption of a glucose-rich diet still led to accumulation of target gene mRNA in adipose tissue. Indeed, hyperglycemia-modulated gene expression has been reported in peripheral tissues [17]. In tissues without GLUT2, glucose detection occurred by GLUT2-independent mechanisms, probably due to “intracellular” metabolism. As expected, these mechanisms were not directly affected by the molecular tool (GLUT2 loop). Accordingly, GLUT2-loop transgenic mice had normal insulin sensitivity in peripheral tissues, mainly muscle and adipose tissue. The capacity of peripheral tissues to respond to insulin remained unaffected by the expression of the transgene.

The amount of GLUT2 protein in the membranes of liver, pancreas and kidney cells showed that the phenotype of transgenic mice was not caused by defective glucose entry into the cells but rather by the inability of the cells to detect extracellular glucose.

A fundamental characteristic of GLUT2-loop transgenic mice was that basal metabolism remained unaffected. Nevertheless, in transgenic mice fed with the usual diet, thermic response occurred but was two times slower than in wild-type mice. Fuel oxidation indicated that transgenic mice utilize more lipids than wild-type mice. Better lipid oxidation improves resistance to high fat feeding [18] and increases weight and fat losses without major changes in lean body mass [19], a feature observed in these transgenic mice.

Wild-type mice use their energy stores during fasting whereas transgenic mice protect their energy stores when deprived of food due to an impaired ability to detect changes in glucose levels. The slower mobilization of glycogen stores in transgenic compared to control mice is probably due to plasma insulin, which remained high during fasting, and to the preference for lipid oxidation over glucose oxidation. Partial preservation of energy stores in transgenic mice occurred despite similar levels of circulating glucose, suggesting that low glucose concentration was inadequately detected in these mice.

Transient protection against streptozotocin-induced diabetes has been observed possibly due to many GLUT2-mediated effects, including drug access to pancreatic ß cells [20], but not to accelerated urinary glucose loss or larger stores of pancreatic insulin.

Therefore, we concluded that GLUT2-expressing tissues involved in glucose homeostasis poorly detected both high and low glucose levels.

### Pancreatic adaptations to impaired detection of extracellular glucose

Pancreatic glucose sensing is mediated by metabolism and primarily by glucokinase activity [3]. How glucokinase activity is translated into a quantitative signal remains unknown. It has been reported that after being metabolized in the ß-cell, glucose stimulates insulin gene transcription by a mechanism involving SREBP-1c [21]. Insulin mRNA accumulated in transgenic mice after consumption of a glucose-rich diet as in control mice. Thus, GLUT2-mediated glucose sensing did not significantly alter transcription and stability of insulin mRNA. Nevertheless, transgenic pancreas had low insulin content, consistent with reports showing that insulin expression is also regulated by glucose at a translational level. Fasted mice had lower islet insulin mRNA content as compared to fed mice [22]. While *in vitro* the acute changes in glucose affected mainly insulin translation [23], [24], [25], [26], chronic changes in glucose impacted on both insulin gene transcription [27] and mRNA stability [28]. In ß-cell, long term glucose sets the preproinsulin mRNA level whereas short term glucose regulates the translation rates of proinsulin biosynthesis [25], [26], [21].

Decreased insulin content in pancreas of transgenic mice may thus result from small ß-cell mass and increased number of small islets compared to controls. Supporting this idea, pancreatic ß-cell expansion is induced by glucose-stimulated proliferation [29]–[32], and glucose-mediated reduction of apoptosis [33], [34]. However, glucotoxicity has been linked to persistent hyperglycemia in type II diabetes as a result of impaired ß-cell proliferation [35], [36] and increased apoptosis [37]. Plasma insulin concentration did not increase adequately in response to a glucose load in GLUT2-loop transgenic mice suggesting that GLUT2-mediated glucose detection is necessary for normal ß-cell development and function. A reduced β cell mass has also been reported in mice, lacking insulin receptors in pancreatic β-cells [38], and in HGFR KO mice [39], suggesting that there are many regulators of pancreatic ß cell mass [40]. Some of these regulators may be under the control of GLUT2-mediated sugar detection. Further investigation with a broader analysis, is necessary to resolve this question.

### Kidney adaptations to impaired detection of extracellular glucose

The urinary excretion of glucose observed here might be due to specific or general tubular adaptations. In the kidney, a failure of tubules to reabsorb small molecules will cause increased urinary excretion of glucose but also of amino acids, minerals, electrolytes (sodium, potassium, bicarbonate) and water as observed in renal Fanconi syndrome [41]. This will result in polyuria, polydipsia, dehydration, hypophosphatemic rickets and growth retardation. Similarly, patients affected by GLUT2 mutation do not reabsorb glucose and various filtered solutes because of impairment in GLUT2-mediated efflux of glucose [42]. Accumulation of glycogen in renal tubular cells has been described in Fanconi-Bickel patients [11]. Glycogen deposit is maintained in the kidney of fasted GLUT2-loop transgenic mice but there was no perturbation of basal kidney function. Such perturbation appeared only after oral glucose load or the consumption of a glucose-rich diet. In GLUT2-loop transgenic mice, normal reabsorption of glucose and associated solutes occurred when blood glucose levels were under 170 mg/dl. Glucose reabsorption is not sufficient in transgenic mice and the threshold is lower than in wild-type mice, but there was no significant electrolyte loss. The renal adaptations related to GLUT2-mediated sugar detection are restricted here to decreased glucose reabsorption. We found a relationship in kidney between glucose detection by GLUT2 and the decrease in SGLT1 mRNA. Mutations in SGLT1 gene provokes glucose-galactose malabsorption accompanied by mild glucosuria whereas familial renal glucosuria is due to mutations in the SGLT2 gene [43]. Several proteins of proximal tubule and their regulators are likely to be involved in the process of glucose tubular reabsorption. Thus, further investigation is required to understand the molecular mechanisms for this renal functional adaptation.

The mouse phenotype described here, characterized by a low level of insulin secretion and a high level of glucosuria, is reminiscent of a monogenic (HNF1α) form of diabetes. MODY3 patients have a defect in insulin secretion but no obvious hyperglycemia probably related to massive urinary glucose loss [44]. The maximal renal reabsorption capacity of these patients, as in HNF1α-null mice, is decreased due to substantially less transcription of a sodium/glucose co-transporter (SGLT2) [45]. In contrast with GLUT2-loop transgenic mice, HNF1α-null mice had defective renal tubular reabsorption of various metabolites (amino acids, phosphates), in addition to glucose, suggesting that HNF1α levels are not involved in the defect we report in mice with an impaired detection of extracellular glucose.

In conclusion, artificial inhibition of GLUT2-mediated sugar sensing in a limited number of tissues, modified various parameters of glucose homeostasis. This may be advantageous because urinary glucose loss can eliminate excess glucose and reduce fat deposits. Moreover, an attenuated secretion of insulin on demand can preserve pancreatic potential in the long term.

## Materials and Methods

### Creation of transgenic mice

The actin promoter was used to drive strong and ubiquitous expression of the transgene. We used pCAGGS-IRES2-EGFP (kindly provided by MR Hirsh), derived from the pCAGGS eukaryotic expression vector [46]. We replaced the internal ribosome entry site (IRES) and the enhanced green fluorescent protein (EGFP) gene with the EGFP-Loop DNA fragment coding for amino acids 237 to 301 previously described [12].

Genotyping of transgenic mice was carried out by PCR with a forward primer recognizing EGFP (5′-CGAATTCCTGTCCAGAAAGCC-3′) and a reverse primer recognizing the GLUT2 loop (5′-AGAAGTCAGATGCTCAAGGGG-3′). The transgene copy number was determined by real-time PCR with the same primers. Apolipoprotein A1 was used as a reference (primers from A. Kalopissis).

The animals were bred in the transgenic animal facilities of IFR58 (Paris). All animal procedures complied with published recommendations for the use of laboratory animals by the French government. Tg B , Tg P and Tg G indicate transgenic mice from the B, P and G lines, respectively.

### Analysis of metabolic parameters

Blood samples were collected from the tail of conscious mice. Blood glucose concentrations were measured with a glucometer (Accu-chek Go, Roche); urinary glucose, with test strips (Keto-Diastix, Bayer); plasma insulin concentrations, with an ELISA kit with a mouse insulin standard (Rat/Mouse insulin Kit, LINCO); plasma free fatty acids (FFA) concentrations, with an NEFA C determination kit (Wako); plasma triglycerides, with a TG enzymatic determination kit (Biomérieux) and tissue glucose content, with a D-glucose/D-fructose determination kit (Boehringer Mannheim/R-Biopharm, Roche).

Levels of urine proteins, creatinine, sodium, potassium, chloride, calcium, phosphate and glucose were measured with an automatic analyzer (Hitachi 911; Boehringer Mannheim).

Tissue glycogen content was measured enzymatically (Roche Applied Science). Before insulin content determination, the pancreas was homogenized in an acid-alcohol solution.

### RNA extraction and quantitative PCR analyses

Total RNA from liver, kidney, pancreas and epididymal fat pads was extracted with Tri-Reagent (MRC). Reverse transcription (RT) was carried out with 1 µg total RNA. Messenger RNA was quantified with the Light-Cycler system (Roche Molecular Biochemicals I primer, Indianapolis). Specific primers were designed for GLUT2 (Fwd: 5′-ACCCTGTTCCTAACCGGG-3′, Rev: 5′-TGAACCAAGGGATTGGACC-3′), proinsulin (Fwd: 5′-AAACCCACCCAGGCTTTTGT-3′, Rev: 5′-ATCCACAATGCCACGCTTCT-3′) SGLT1 (Fwd: 5′-GGGTGGCTTTGAATGGAA-3′, Rev: 5′-CCTTGATGTAAATCGGGACAA-3′) and SGLT2 (Fwd: 5′-GCTGGATTTGAGTGGAATGC-3′, Rev: 5′-CGGTCAGATACACTGGCACA-3′). Primers used to detect glucokinase, SREBP-1c and ChREBP [47], FAS and ACC [48] have been described previously. L19 was used as an internal control [49].

### Immunoblotting analysis and immunoprecipitation

Crude membrane preparations were obtained as previously described [50]. The membrane proteins were resolved by 12% SDS-PAGE and transferred to nitrocellulose membrane.

Membrane proteins were analyzed by immunoblotting with antibody against the GLUT2 intracellular loop (produced by Eurogentec) and the first GLUT2 extracellular loop (Chemicon).

For immunoprecipitation, frozen liver was homogenized in RIPA buffer (20 mM Tris pH7.5, 1 mM, EDTA, 0.15M NaC1, 10 mM KC1, 1%NP-40, 0.1% deoxycholate, 0.1% SDS) containing protease inhibitor cocktail (Roche) and 42 mM MG132 (Calbiochem). Liver extracts were incubated with rabbit polyclonal antibody against the GLUT2 intracellular loop and protein G-Sepharose beads. The immune complexes were analyzed by immunoblotting with the antibody against the GLUT2 loop.

### Immunohistochemical and morphometric analyses

The pancreas was fixed overnight in Bouin's solution and embedded in paraffin according to standard procedures. Ten sections per pancreas, taken every 72^nd^ serial section (6 µm thick) obtained throughout the block of pancreas, were immunostained for insulin (guinea pig anti-porcine insulin antibody, 1∶500, ImmunO, MP biomedical), detected with a peroxidase-conjugated rabbit anti-guinea pig antibody (1∶50, Dako) and a peroxidase substrate kit (DAB, Vector Laboratories). Tissue sections were counterstained with hematoxylin. Morphometrical analyses were performed as described in [51]. Surface area occupied by insulin staining and total pancreatic tissue were quantified with a 10× objective on a Leica microscope outfitted with a camera and the Leica Qwin software (Leica, France). The §-cell fraction (percent beta-cell in the pancreas) represents the ratio of insulin-positive cell area to the total pancreatic tissue area on the entire section. §-cell mass was calculated as the surface ratio between §-cell and total pancreatic tissue multiplied by pancreatic weight. The proportions of small (<25 µm), medium (>26 to <100µm), and large (>100µm) islets were also quantified. A total of 250-500 islets were counted per pancreas. At least 3 mice were analyzed per group.

Histological sections of pancreas were incubated with Schiff reagent (Merck) for glycogen visualization.

### *In vivo* studies

Mice were fed either a standard carbohydrate diet (23% proteins, 51% carbohydrates, 3% lipids) obtained from Dietex (Saint-Gratien, France) or a glucose-rich diet (19% casein, 0.3% methionine, 65% dextrose) as previously described [52].

For oral glucose tolerance tests (OGTT), mice were fasted for 24 h and then received a glucose load of 3.6 g/kg. For insulin tolerance tests (ITT), mice were injected intraperitoneally with 1unit/kg insulin (Actrapid Novo).

When required, mice were injected with 200 mg/kg streptozotocin (Sigma).

For urine analysis, mice were individually housed in metabolic cages with free access to water and a standard or glucose-rich diet. The mice were allowed to adapt to the cages for three days. Intake of food and water was then measured every 24 h and urine was collected throughout three consecutive days. Spontaneously excreted urine from each 24 h-period was collected for measurement of urinary creatinine and protein excretion.

### Indirect calorimetry

These studies were conducted in an open-circuit, indirect calorimetric device [53], [54]. The apparatus was improved and developed within the framework of the AddenFi project, for the valorization and commercialization of laboratory setups, at INRA-AgroParisTech, INA-PG UMR 914 Research Unit [53]. The temperature in the metabolic cage was maintained at 32±1°C. Oxygen consumption and carbon dioxide production were recorded every 10 s using a computer-assisted data acquisition program written under Labview 7.1. A previously described stoichiometric formula was used to calculate oxidation of glucose and lipid. Protein oxidation was not taken into account. Resting and activity-related metabolic rates were calculated separately (Kalman filtering method) [53], [54], [55].

The mice were placed in the metabolic cage at 9:00 a.m. with water, but no food, available. An oral glucose tolerance test (3.6 mg/g) was carried out at 2:00 p.m. and a calibrated test meal (1g of the standard diet) was given at 6:00 p.m. Respiratory exchanges and spontaneous activity were continuously recorded until 9:00 a.m. the following day.

The basal metabolic rate was defined as the mean resting metabolic rate measured between 5:00 a.m. and 7:30 a.m. when the mice were in a post-absorptive state. The changes in metabolic rate, respiratory quotient, glucose and lipid oxidations induced by the OGTT and test meal were calculated relative to the stable pre-test values measured during the preceding hour.

### Total fat

Total fat was determined by dual-energy X-ray absorptiometry (Lunar PIXImus mouse densitometer; GE LUNAR Corp.). Mice were fasted for 48h before the analysis and anesthetized with Avertin (0.02 ml/g body weight).

### Kidney echography

Ultrasonic images were acquired *in vivo* using a clinical dermatological imaging system (Ultrasons Technologies, Tours, France) with custom modifications for dynamic data acquisition and high-frequency electronics [56]. Mice were anesthetized with 1.5% isoflurane.

### Statistical analyses

Results are presented as means±S.E.M., and unpaired *t* test was used for data analysis unless indicated (i.e. ANOVA) (GraphPad Software).
